# Associations of EAT-*Lancet* Planetary Health Diet or Finnish Nutrition Recommendations with changes in obesity measures: a follow-up study in adults

**DOI:** 10.29219/fnr.v67.9107

**Published:** 2023-12-01

**Authors:** Tiina Suikki, Mirkka Maukonen, Niina E. Kaartinen, Kennet Harald, Sari Bäck, Laura Sares-Jäske, Tommi Härkänen, Seppo Koskinen, Pekka Jousilahti, Anne-Maria Pajari, Satu Männistö

**Affiliations:** 1 Finnish Institute for Health and Welfare, Department of Public Health and Welfare, Helsinki, Finland; 2 University of Helsinki, Department of Food and Nutrition, Helsinki, Finland

**Keywords:** dietary index, overall diet quality, food-based dietary guidelines, sustainable diet, sustainability, weight changes

## Abstract

**Background:**

Knowledge on the association between the EAT-*Lancet* Planetary Health Diet (PHD) or the Finnish Nutrition recommendations (FNR) and anthropometric changes is scarce. Especially, the role of the overall diet quality, distinct from energy intake, on weight changes needs further examination.

**Objectives:**

To examine the association between diet quality and weight change indicators and to develop a dietary index based on the PHD adapted for the Finnish food culture.

**Methods:**

The study population consisted of participants of two Finnish population-based studies (*n* = 4,371, 56% of women, aged 30−74 years at baseline). Dietary habits at the baseline were assessed with a validated food frequency questionnaire including 128−130 food items. We developed a Planetary Health Diet Score (PHDS) (including 13 components) and updated the pre-existing Recommended Finnish Diet Score (uRFDS) (including nine components) with energy density values to measure overall diet quality. Weight, height, and waist circumference (WC), and the body mass index (BMI) were measured at the baseline and follow-up, and their percentual changes during a 7-year follow-up were calculated. Two-staged random effects linear regression was used to evaluate β-estimates with 95% confidence intervals.

**Results:**

Adherence to both indices was relatively low (PHDS: mean 3.6 points (standard deviation [SD] 1.2) in the range of 0−13; uRFDS: mean 12.7 points (SD 3.9) in the range of 0−27). We did not find statistically significant associations between either of the dietary indices and anthropometric changes during the follow-up (PHDS, weight: β −0.04 (95% CI −0.19, 0.11), BMI: β 0.05 (−0.20, 0.10), WC: β −0.08 (−0.22, 0.06); uRFDS, weight: β 0.01 (−0.04, 0.06), BMI: β 0.01 (−0.04, 0.06), WC: β −0.02 (−0.07, 0.03)).

**Conclusion:**

No associations between overall diet quality and anthropometric changes were found, which may be at least partly explained by low adherence to the PHD and the FNR in the Finnish adult population.

## Popular scientific summary

- Further research of the recently published EAT-*Lancet* Planetary Health Diet in the context of different food cultures and health outcomes is required. We developed a dietary index to measure adherence to the planetary health diet in the Finnish adult population independently of energy intake.
- In this study, diet quality was not associated with anthropometric changes during a 7-year follow-up.
- Low adherence to diets, however, may attenuate the associations between diet quality and health outcomes.

Overweight and obesity − chronic multifactorial metabolic conditions characterized by excessive fat accumulation − have reached epidemic proportions. Globally, in 2016, 39% of adults (aged 18 years and above) were living with overweight (body mass index [BMI]: 25−29 kg/m^2^) and 13% with obesity (BMI 30 kg/m^2^ and over) (1). However, it is estimated that already by 2030, 17.5% of adults will be living with obesity (2). In Finland, 72% of men and 63% of women were living with overweight, and 25% of both men and women were living with obesity in 2017 (3). By 2040, it is estimated that 29% of men and 27% of women will be living with obesity in Finland (4). Obesity increases the risk of non-communicable diseases, for example, type 2 diabetes, cardiovascular diseases, and certain types of cancers, and premature mortality (2). Furthermore, the consumption of food over physiological needs, also referred as ‘metabolic food waste’, represents a burden to the environment because of, for example, unnecessary greenhouse gas emissions due to the over-production of food (5).

The fundamental cause of weight gain is a long-term imbalance between energy intake and expenditure. However, specific foods and food groups such as refined grains, red and processed meat, and sugar-sweetened beverages are possible mediators suggested in the recent systemic review and meta-analysis of 43 reports based on 25 prospective studies (6). This could be explained by increased probability of a positive energy balance concerning intake of specific food groups. This can further contribute to changes in insulin secretion and resistance as well as regulation of hunger and satiety in which the macronutrient composition of the diet in general is critical (6).

Despite the mediating role of single foods and nutrients on weight gain, they may also have cumulative effects on health, and, thus, whole-diet approach could be more beneficial compared to looking at individual foods or nutrients (7). National food-based dietary guidelines (FBDGs) are an example of a whole-diet approach to promote overall health. For example, in a systematic review of observational studies (*n* = 34) conducted from 1990 to 2016 in adults worldwide, and in a systematic review of observational and experimental studies (*n* = 38) conducted from 2015 to 2020 in adults worldwide, higher adherence to dietary guidelines has been associated with a lower risk for obesity (8, 9). In Finland, an analysis of cross-sectional data has shown that higher adherence to the Finnish Nutrition Recommendations (FNR) (including food-based and nutrient-level guidelines) in adults (*n* = 4,720) was inversely associated with waist circumference (WC) and body fat percentage (10).

Additionally, a global modeling study showed that better adherence to FBDGs improved both human and environment health on the global and local levels. However, the national FBDGs could be even healthier and more environmentally sustainable (11). In 2019, the EAT-*Lancet* Commission launched a set of global dietary recommendations as a reference diet, referred as ‘a Planetary Health Diet’ (PHD), to enable global scientific targets for a healthy diet obtained from sustainable food systems (12). Based on a modeling study, the adoption of the PHD could reduce premature mortality by 34% more than adoption of the national FBDGs only (11). In addition, adoption of the PHD was associated with reduced proportion of overweight and obesity. After the release of the PHD, the association between the PHD and anthropometric measures, however, has been examined in adults only cross-sectionally, and the results have been conflicting (13–18).

Thus far, there are no studies examining the association between the adherence to the PHD or the FNR independently of energy intake and weight changes. Therefore, the aim of our study was to examine whether overall diet quality based on the PHD or the FNR could predict anthropometric changes during a 7-year follow-up period. We developed a dietary index based on the PHD adapted to Finnish food culture and updated the pre-existing dietary index for the FNR (10). We expected that a higher adherence to both dietary recommendations, distinct from energy intake, at the baseline would be associated with stability in anthropometric measures during the follow-up period.

## Methods

### Study population

We used participants from two Finnish population-based health examination studies, the Health 2000 Study (19) and the DIetary Lifestyle and Genetic Determinant of Obesity and Metabolic Syndrome (DILGOM) Study 2007 (20), and their follow-ups, Health 2011 (21) and DILGOM 2014 (22), both conducted by the Finnish Institute for Health and Welfare (THL). The purpose of the Health 2000 and 2011 studies was to examine the most crucial public health problems, their causes and treatments, and population’s functional and working capacity in adults (aged 30 and over at baseline) (19, 21). Of the original study sample (*n* = 8,028), 84% (*n* = 6,771) participated in a health examination and interviews in 2000, and 63% of the invited (*n* = 6,319) took part in the health examination of the follow-up study in 2011 (*n* = 4,006) (Supplemental Fig. 1). DILGOM 2007 and 2014, a sub-study of the National FINRISK 2007 Health Study (23), aimed to obtain more specific information of obesity risk factors and metabolic syndrome in adults (aged from 25 to 74 years at baseline) (20). All FINRISK 2007 participants (*n* = 6,258) were invited to DILGOM 2007, of which 80% (*n* = 5,024) participated in a health examination. In 2014, 82% of the invited (*n* = 4,581) participated in the follow-up (Supplemental Fig. 1) (20, 22). The studies are described in detail elsewhere (19–23).

All studies included health examinations with anthropometric measurements and self-administered questionnaires (with questions on the sociodemographic background, lifestyle, and overall diet). For the present study, all participants with a baseline food frequency questionnaire (FFQ) and baseline and follow-up information on weight, height, and WC measured by trained research nurses were included. Furthermore, we harmonized both cohort studies based on age so that participants who were ≥30 years old at the baseline and ≤81 years old at the follow-up were included (excluding 237 participants). Pregnant women at the baseline or follow-up were also excluded (*n* = 57). Furthermore, participants whose daily energy intake corresponded to 0.5% at either end of the energy intake distribution were excluded from the DILGOM 2007 study (*n* = 48) (24), and participants with daily energy intake values <600 and >7,000 kcal were excluded from the Health 2000 study (*n* = 18). The analytical study sample for this study comprised 4,624 participants (Health 2000/2011, *n* = 3,432 (51% of 6,771 participants attended the health examination at the baseline); DILGOM 2007/2014, *n* = 1,192 (24% of 5,024 participants attended the health examination at the baseline)).

All studies were conducted according to the guidelines of the Declaration of Helsinki, and the research protocols were approved by the Ethics Committee of the Hospital District of Helsinki and Uusimaa. A written informed consent was obtained from all participants.

### Dietary intake and dietary indices

The FFQ inquired about the participants’ habitual diet over the last 12 months. The FFQ was originally designed in 1996 and has been updated and repeatedly validated against food records in the Finnish adult populations (25–27). The FFQ was filled in at the study site or at home and asked to be sent by mail to THL.

The frequency of consuming each food item (128–130 depending on the study) in the FFQ was reported in nine categories (‘never or seldom’, ‘1–3 times a month’, ‘once a week’, ‘2–4 times a week’, ‘5–6 times a week’, ‘once a day’, ‘2–3 times a day’, ‘4–5 times a day’ or ‘six or more times a day’). The portion size for each FFQ-item was fixed and appeared on the FFQ as natural units (e.g. slice or glass). The average daily consumption of foods (ingredient level) and intakes of nutrients and energy were calculated using the FINESSI in-house software, which utilizes the Finnish National Food Composition Database (Fineli^®^) (28).

The diet quality was assessed with two dietary indices based on the PHD (12) and the FNR (29).

#### Planetary Health Diet Score (PHDS)

As the PHD has been introduced as a global reference diet which should be modified for each food culture, we composed a PHDS dietary index for Finnish food culture (12). All original components (whole grains [including rice, wheat, corn, and other], tubers or starchy vegetables, vegetables, fruits, dairy foods, beef and lamb, pork, chicken and other poultry, eggs, fish, legumes [including dry beans, lentils and peas, soy foods, and peanuts], tree nuts, added fats [including palm oil, unsaturated oils, dairy fats, lard, or tallow], and all sweeteners) are included in the PHDS, except for ‘added fats’ as daily mean intakes in grams. Instead, we used the ratio of unsaturated and saturated fat intake to refer to the quality of fat intake (Table 1, Supplemental Table 1). Furthermore, we used rye, oats, and barley to reflect whole grains (30), and nuts and seeds as a category to reflect the intake of peanuts and nuts in the Finnish diet. Dairy foods included all the liquid milk products and other dairy products, for example, cheese and cream, as milk equivalents (cheese in grams was multiplied with 5, cream with 2.7, and butter with 6.5). The developed PHDS included 13 components.

**Table 1:** Dietary index variables included in Planetary Health Diet Score (PHDS) adapted for Finnish food culture and updated Recommended Finnish Diet Score (uRFDS)

PHDS	uRFDS
**Whole grains** *Rye, oats, and barley*	**Whole grains** *Rye, oats, and barley*
**Vegetables** *Leafy vegetables, fruit vegetables, cabbages, mushrooms, legumes, and roots (excluding potato)*	**Vegetables** *Leafy vegetables, fruit vegetables, cabbages, mushrooms, legumes, roots (excluding potato), and nuts and seeds*
**Potatoes**	
**Fruits and berries** *Apples, citruses, and other fruits and berries*	**Fruits and berries** *Apples, citruses, and other fruits and berries*
**Dairy foods** All milks, sour milk, and cream, butter and cheese as milk equivalents*	
**Red and processed meat** *Beef, pork, lamb, sausage, meat products, and offal*	**Red and processed meat** *Beef, pork, lamb, sausage, meat products, offal, and game*
**Chicken and other poultry**	
**Eggs**	
**Fish and fish products** *Including shellfish*	**Fish and fish products** *Including shellfish*
**Legumes** *Beans, lentils, peas, and soy products*	
**Nuts and seeds**	
**Ratio of PUFA to SFA+trans-fatty acids**	**Ratio of PUFA to SFA+trans-fatty acids**
**Sucrose** *g/day*	**Sucrose** *g/day*
	**Salt** *g/day*
	**Alcohol** *g/day*

* Based on Stockholm Resilience Centre evaluations (Wood et al. (47), erratum.

PUFA, polyunsaturated fatty acids; SFA, saturated fatty acids; g/d, grams/day.

The EAT-*Lancet* PHD provides reference intake values for food groups per day presuming a daily energy intake of 2,500 kilocalories (kcal) (12). Therefore, the PHDS was developed to refer intakes of all food groups together if the participant consumed 2,500 kcal/day. First, we divided the participants’ absolute daily consumption of each index component as grams by the total daily energy intake of the participant as kilocalories (excluding the fat ratio component). Then, the grams per kcal-ratios were multiplied by 2,500. The components were coded based on the cut-off values: 0 points when not meeting the chosen cut-off value or 1 point when meeting the cut-off value (Supplemental Table 1). The total score could range from 0 to 13 points. The higher total score indicates better adherence to the PHD.

The cut-off values were based either on the lower, upper, or mean daily consumption target levels of the PHD (12). When choosing between the lower, upper, or mean target level, the following aspects were considered: healthiness (‘protective’, ‘neutral’, or ‘limit’ (31)), and the environmental impacts (‘low’, ‘medium’, or ‘high’ (32)) of the component, the importance of the component in the Finnish food culture, and the methodological aspects such as the tendency of FFQs to overestimate the consumption of some foods, for example vegetables and fruits (25) (Supplemental Table 1). For example, for fish consumption, the upper limit of the given range by EAT-*Lancet* Commission (≤100 g/day) was chosen for cut-off value in the index, as especially freshwater fish with relatively low environmental impact is emphasized in the Finnish food culture.

#### Updated Recommended Finnish Diet Score

We updated the original Recommended Finnish Diet Score (10), which was based on the FNR 2004, to measure the adherence to the current recommendations from 2014 (29). The Updated Recommended Finnish Diet Score (uRFDS) included nine variables (Table 1). Compared to the original index, we added one food group variable (fish and fish products), extended the rye group with oats and barley to refer better to whole grains in the Finnish diet (30) and replaced the white and red meat ratio with the food group of red and processed meat.

To analyze the diet quality distinctly from the energy intake more precisely, we standardized the food consumption for the energy intake by dividing the participant’s mean daily intakes of each index components in grams by the total mean daily energy intake of the participant (except the fat ratio). Then, points were given based on the quartiles of consumption as grams per energy unit of each component (Supplemental Table 2). For whole grains, vegetables, fruits and berries, fish, and fat ratio, the lowest quartile of intake was coded as 0 points, the second as 1 point, the third as 2 points, and the highest quartile of intake as 3 points. For red and processed meat, salt, sucrose, and alcohol, the scoring was opposite. Thus, the total score could range from 0 to 27 points, where a higher total score indicated better adherence to the FNR 2014.

### Anthropometric variables

At both the baseline and follow-up, the weight, height, and WC were measured by trained research nurses at the study sites according to standardized international protocols with the participants wearing light clothing and no shoes (33). The height was measured with a wall-mounted or stand-alone stadiometer to the nearest 0.1 or 0.5 cm. The weight was measured with a beam balance scale or as a part of the bioimpedance analysis to the nearest 0.1 or 0.5 kg. The BMI was calculated as weight (kg) divided by the squared height (m^2^). The WC was measured with a flexible, non-elastic measuring tape from the mid-point of the lowest rib bones and iliac crest to the nearest 0.1 or 0.5 cm.

The absolute change in the weight, BMI, and WC during the follow-up was calculated as follows: follow-up measurement – baseline measurement, where positive values indicated weight gain and negative values indicated weight loss. The percentual change was calculated as the absolute weight, BMI, or WC change during the follow-up divided by baseline measure of weight, BMI, or WC. Percentual weight, BMI, and WC changes were further categorized into four groups: ≤−5.0% (weight loss), −4.9% to +4.9% (weight stable), +5.0% to +9.9% (moderate weight gain), and ≥+10.0% (substantial weight gain).

### Sociodemographic and lifestyle factors

Sociodemographic and lifestyle factors, for example, education, leisure-time physical activity, and smoking status, were assessed using self-administered questionnaires. Education was harmonized to three categories: low (did not graduate from upper secondary school or vocational school or was in the lowest tertile of the total number of school years according to sex and birth cohort to adjust for the extension of the basic education system and increase in average school years over the last decades), medium (graduated from upper secondary school or was in the middle tertile), and high (graduated from university or university of applied sciences or was in the highest tertile). Leisure-time physical activity comprising the activity outside work was harmonized into three groups: inactive (light activities, like reading and watching television), moderately active (such as walking, cycling, and gardening at least 4 h/week), and active or very active (brisk running, swimming, or other physically demanding activities at least 3 h/week or competition sports). Smoking habits were classified into three categories: never smokers, former smokers, and current smokers.

### Statistical methods

Men and women were combined in the analyses, since no significant interaction was found between the sexes when the association was analyzed between diet quality and anthropometric measures. Follow-up years were standardized to 7 years between the studies because of the different follow-up periods of the included datasets (7 and 11 years). Anthropometric changes during the 11 follow-up years of the Health 2000 studies were first divided by 11 and then multiplied by 7 to harmonize it with the follow-up period of the DILGOM study.

The baseline characteristics of the participants for the studies are presented as means with standard deviations for continuous variables or as proportions for categorical variables. Pooled descriptive statistics are presented by anthropometric change categories separately for weight, BMI, and WC. Furthermore, pooled intakes of carbohydrates, protein, and fat as percentual intake of total energy intake, energy-standardized fiber intake, and each PHDS component by the PHDS scoring groups are presented as means with standard deviations. Mean dietary index scores with standard deviations are also presented by sex (men or women), education level (high or low), and smoking status (current smoker and non-smoker). Variables that did not satisfy the normality assumption were log transformed with the natural logarithm.

A linear regression was used to calculate study-specific β-estimates and 95% confidence intervals (CI) for each dietary index (PHDS and uRFDS) at the baseline as a continuous exposure variable, and baseline anthropometric measures (weight, BMI, and WC) and percentual anthropometric changes (weight, BMI, and WC) as continuous outcome variables. Then, study-specific β-estimates and 95% CI with *P*-values were pooled with a two-staged random effects linear regression (34). The heterogeneity was tested between the pooled cohorts using Q-statistics. Furthermore, to evaluate the plausibility of the PHDS and uRFDS in relation to its components, the pooled *P*-value for the trend in the association between the intake of each of the uRFDS or PHDS components and the scoring groups was determined with a two-staged random effects linear regression (34).

We used two main models in the analyses and further carried out a sensitivity analysis. The first model (model 1) was adjusted for age, sex, and the baseline anthropometric measure (weight, BMI, and WC) depending on which of these was examined. In addition, the weight change was adjusted with the participant’s baseline height information. In the second model (model 2), model 1 was further adjusted for the baseline information on education, leisure-time physical activity, and smoking status. Additionally, model 2 was further adjusted for the participant’s baseline total energy intake to observe the effect of energy residues on the results, even though the energy intake was already considered when both dietary indices were composed. In the first sensitivity analysis, we excluded from the model 2 all participants reporting back pain, arthritis, cancer, diabetes, and depression or any other mental illness (*n* = 724) as these conditions are commonly known medical conditions linked to weight changes (35, 36). In the second sensitivity analysis, we excluded participants with energy under-reporting from model 2 (*n* = 1,221). The analysis of energy under-reporting was based on the calculation of ratio of reported energy intake and predicted basal metabolic rate, where the ratio ≤1.14 was considered to indicate energy under-reporting (37, 38).

Statistical analyses were conducted using SPSS statistical computing software version 27.0 (IBM SPSS Statistics), and the pooling of the cohorts was carried out with the R statistical computing program, version 4.1.1, package meta (39). A *P*-value of less than 0.05 was considered as statistically significant.

## Results

The pooled data included 4,371 participants (56% women). The mean age across the studies at the baseline was 49 years (Table 2). The participants were more often highly educated, one-fifth were current smokers, and one-fifth were inactive in their leisure-time. The mean BMI and WC in men were 27 kg/m^2^ and 97 cm, respectively. For women, the mean BMI was 26 kg/m^2^, and the mean WC was 86 cm. Furthermore, participants in the highest weight gain (incl. BMI and WC) categories (≥+10%) tended to be more often women, younger, highly educated, current smokers, and physically inactive in their leisure-time compared to participants in the weight loss or weight stable categories (Supplemental Table 3).

**Table 2:** Baseline characteristics of participants by studies and in total presented as means with standard deviations or percentages

	Health 2000	DILGOM 2007	All
Number of participants^1^	3,432	1,192	4,624
Age, years	47.8 (10.6)	54.2 (11.6)	49.5 (11.2)
Women, %	55	56	55
Education^2^, %			
High	36	47	39
Low	28	20	26
Smoking habits, %			
Current smoker	26	15	23
Never smoked	53	58	54
Leisure-time physical activity^3^, %			
Inactive	22	18	21
Active/very active	21	29	23
Energy, kcal	2,272 (747)	2,406 (867)	2,306 (781)
Index scores^4^			
PHDS	3.5 (1.1)	4.0 (1.3)	3.6 (1.2)
uRFDS	13.5 (3.8)	13.5 (3.8)	13.5 (3.8)
Height, cm			
Men	177.1 (6.6)	176.6 (6.6)	177.0 (6.6)
Women	163.4 (6.2)	163.7 (6.0)	163.5 (6.1)
Weight, kg			
Men	84.7 (13.2)	83.6 (12.1)	84.4 (12.9)
Women	69.8 (13.1)	70.6 (13.5)	70.0 (13.2)
BMI, kg/m^2^			
Men	26.9 (3.8)	26.8 (3.6)	26.9 (3.7)
Women	26.2 (4.9)	26.4 (5.2)	26.2 (4.9)
WC, cm			
Men	97.1 (10.8)	96.6 (10.7)	96.9 (10.7)
Women	86.3 (12.5)	86.9 (13.2)	86.5 (12.7)

1 All participants with information on food frequency questionnaire and anthropometric measurement at baseline and follow-up were included in the current study.

2 Health 2000: high (graduated from university or university of applied sciences) or low (did not graduate from upper secondary school or vocational school); DILGOM: in the lowest or highest tertile of the total number of school years according to sex and birth cohort to adjust for the extension of the basic education system and increase in average school years over the last decades.

3 Leisure-time physical activity: inactive (light activities, like reading and watching television) or active or very active (brisk running, swimming, or other physically demanding activities at least 3 h/week or competition sports).

4 PHDS ranged from 0 to 13 points, and uRFDS ranged from 0 to 27 points.

RFDS, Recommended Finnish Diet Score; PHDS, Planetary Health Diet Score; WC, waist circumference.

**Fig. 1:**
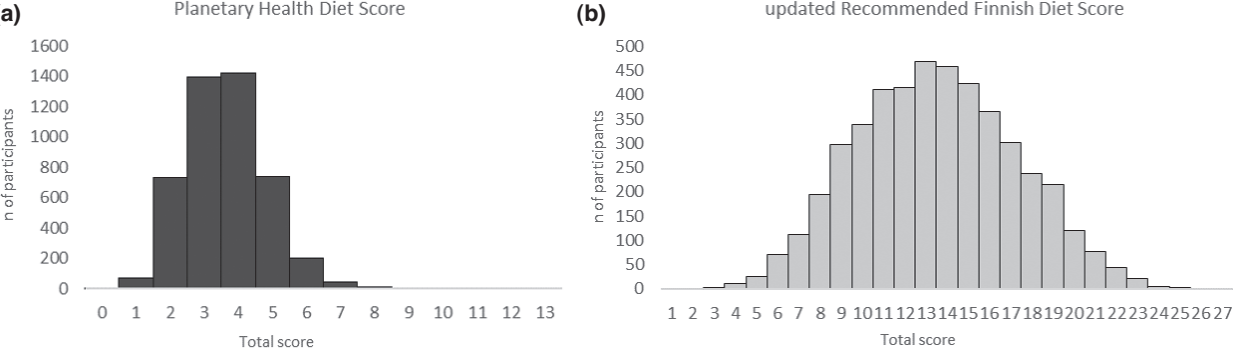
Baseline distribution of total scores of dietary indices in the pooled data (*n* = 4,624). (a) Planetary Health Diet Score. Total score ranged from 0 to 11 points when theoretical maximum could have been 13 points (mean 3.6 points, SD 1.2). (b) Updated Recommended Finnish Diet Score. Total score ranged from 3 to 25 points when theoretical maximum could have been 27 points (mean 13.5 points, SD 3.8).

In the pooled dataset, the mean score for PHDS was 3.6 (SD 1.2) ranging from 0 to 11 points (the highest 10 and 11 points were received by only one participant each) when theoretical maximum could have been 13 points (Fig. 1). Furthermore, the mean total score did not differ between men and women, highly or lower educated participants, or current smokers and non-smokers (Supplemental Table 5). For the PHDS components of whole grains, red and processed meat, and sucrose, the scoring cut-off values (≥ 232 g, ≤ 28 g, and ≤ 31 g as a mean daily consumption when presuming a daily intake of 2,500 kcal, respectively) were not reached in any of the PHDS point groups (0−9 points) (Supplemental Table 4). However, all the PHDS components and nutrients were associated with the PHDS statistically significantly (*P* < 0.05) except the protein intake (*P* = 0.11) and the sucrose component (*P* = 0.946) (Supplemental Table 4). For example, the mean daily intake of carbohydrates and fiber and the consumption of vegetables, fruits and berries, and legumes were positively associated with the PHDS, whereas the mean daily intake of fat and the mean daily consumption of red and processed meat, poultry, and dairy foods were inversely associated. Furthermore, the mean daily consumption of fish and fish products reached the cut-off value in almost every total score group (1−9 points); however, the mean daily consumption decreased by a higher total score (*P* < 0.05).

Furthermore, the uRFDS ranged from 3 to 25 points (maximum of 27 points) with a mean score of 13.5 points (SD 3.8) (Fig. 1). For the uRFDS values, the findings were similar for the index components by total scores as in the PHDS except that the sucrose component was also statistically significantly associated with the uRFDS (*P* < 0.05) (data not shown). For example, the mean daily consumption of whole grains, vegetables, fruits and berries, and fish and fish products was positively associated with the uRFDS, whereas the mean daily consumption of red and processed meat, alcohol, and sucrose was inversely associated.

The diet quality evaluated with both dietary indices (PHDS and uRFDS) was negatively associated with the baseline WC measures in the pooled datasets in model 2 (PHDS: β −0.004 (95% CI −0.007, −0.000), *P* = 0.03; uRFDS: β −0.002 (−0.003, 0.000), *P* = 0.03) but not with the baseline weight (PHDS, *P* = 0.08; uRFDS, *P* = 0.48) or the BMI (PHDS, *P* = 0.12; uRFDS, *P* = 0.48). However, there were no statistically significant associations between either of the two dietary indices and changes in weight, BMI, or WC during the 7-year follow-up (*P* > 0.05) except for a negative significant association between percentual change in WC and uRFDS (β −0.054 (−0.100, −0.009), *P* = 0.02, model 1) (Table 3). However, this finding was attenuated in model 2. Furthermore, no significant between-study heterogeneity was found in any of the analyses (*P* > 0.05).

**Table 3:** Pooled linear association as β with 95% confidence intervals and *P*-values between dietary indices at baseline and change in anthropometrics during follow-up

	Planetary Health Diet Score			Updated Recommended Finnish Diet Score		
	β (CI 95%)	*P*_value_ ^1^	*P*_het_ ^2^	β (CI 95%)	*P*_value_ ^1^	*P*_het_ ^2^
**Weight change, %**						
Model 1	−0.03	0.74	0.28	−0.02	0.42	0.28
	(−0.19; 0.13)			(−0.08; 0.03)		
Model 2	0.02	0.98	0.33	0.01	0.86	0.38
	(−0.14; 0.15)			(−0.05; 0.05)		
**BMI change, %**						
Model 1	−0.05	0.56	0.28	−0.03	0.33	0.27
	(−0.21; 0.11)			(−0.09; 0.03)		
Model 2	−0.003	0.89	0.33	0.004	0.88	0.35
	(−0.15; 0.13)			(−0.05; 0.05)		
**WC change, %**						
Model 1	−0.07	0.28	0.62	−0.05	**0.02**	0.52
	(−0.21; 0.06)			(−0.10; −0.01)		
Model 2	−0.05	0.45	0.59	−0.03	0.22	0.44
	(−0.18; 0.08)			(−0.08; 0.01)		

Anthropometric changes were harmonized between the studies by dividing the change by the follow-up years (7 or 11) and then multiplied by 7 to standardize changes.

1 *P*-value for linear association. Significance level was at 0.05.

2 Two-sided *P*-value for heterogeneity between studies (Q statistic).

Model 1 is adjusted for sex, age, and further with log-transformed baseline weight/BMI/WC depending on which of these was examined. Weight change was additionally adjusted for log-transformed baseline height.

Model 2 is Model 1 further adjusted for education, smoking, and leisure-time physical activity.

Moreover, the results remained similar after further adjustment of model 2 for the total energy intake or exclusion of energy under-reporters or participants reporting any of the possible confounding health problems from the data (data not shown).

## Discussion

This is the first longitudinal study in adults to examine the association between overall diet quality, independently of energy intake, evaluated with two different dietary indices based on PHD or FNR 2014 and anthropometric changes. In general, the adherence to a healthy diet assessed with both dietary indices was relatively low. No association was found between either of the dietary indices and the anthropometric measures at the baseline or relative anthropometric changes in the standardized 7 years of follow-up in the Finnish adult population.

### Dietary indices and changes in anthropometrics

No previous longitudinal studies exist of the association between the PHD and anthropometric measures in adults, and findings in cross-sectional studies have been conflicting (13–18). In most of the studies, energy intake has not been included in the dietary index to assess the adherence to the PHD. Only in the study by Cacau et al. (40), the energy density was considered in the index scoring by calculating each index component’s energy contribution to the reference diet. Based on this index with a total score of 0–150 points, they found an inverse linear association between the PHD and BMI and WC among Brazilians (*n* = 14,151 men and women, aged 35−74 years) (14).

Previously, the association between FNR and anthropometric measures in the general adult population has been examined in only one cross-sectional study (*n* = 4,720) (10). It was found that better adherence to the FNR 2004 assessed with the original RFDS was likely to maintain a healthy WC and body fat percentage, but not BMI, while adjusting statistical analyses for energy intake. Furthermore, as the FNR is based on the Nordic nutrition recommendations, the association has been studied more comprehensively between the Nordic diet and anthropometric measures. For example, a systematic review and meta-analysis of seven randomized controlled clinical trials in adults found a positive effect of better adherence to the Nordic diet on weight loss over the time ranging from 1.5 to 6 months (41). Furthermore, a prospective study examining the association between the healthy Nordic diet assessed with the Baltic Sea Diet score and anthropometric changes (in DILGOM 2007 and 2014 studies, *n* = 3,067) found an association between better adherence to the healthy Nordic diet at the baseline and decreased weight and BMI during a 7-year follow-up (20). The divergences in consideration of the energy intake in constructing indices between the updated RFDS and Baltic Sea Diet Score could have affected the different results compared to the current study.

Energy balance is an important factor to be considered when studying weight changes and weight maintenance. In the current study, however, the aim was specially to assess diet quality distinctly from the energy intake. Therefore, the energy intake was considered already during developing and updating both dietary indices. In the PHD, recommendations are standardized for a specific energy intake level (2,500 kcal/day) to target overconsumption considering both human health and environmental aspects (12). In other words, the EAT-*Lancet* Commission expressed their recommendations as energy density values. In contrast to the PHD, the FNR 2014 emphasizes the importance of adequate energy intake based on a basal metabolic rate and moderate physical activity without the specific common energy intake recommendations included in FBDG (29). Furthermore, the purpose of the recommendations is not to promote weight loss but healthy weight maintenance. These differences in energy considerations in the recommendations led to different methods to construct the dietary indices in the current study. Overall, our findings could be explained by the fact that energy balance is one of the key factors in weight management, and excluding the energy intake could have attenuated the findings.

However, another explanation for our findings in addition to the energy intake considerations could be the very low adherence to both indices, especially to the PHDS, with a narrow score range which cannot distinguish participants enough from each other. This was also seen when the mean total score of PHDS was compared between men and women, high and low educated participants, and current smokers and non-smokers. The PHD scores did not differ although it is known that women, higher educated and non-smokers, have better diet quality in general. Furthermore, a Swedish study examining the association between adherence to the PHD, with a dietary index without energy consideration, and mortality (*n* = 22,421) observed low adherence to the index with a narrow score range, which complicated distributing the participants to groups based on the index scores (15). Furthermore, in the early 2000s, when our datasets were collected, the environmental aspects of diets were not that familiar as they may be nowadays, which could have also affected low the adherence especially to the PHD. Therefore, continuous data collection is warranted to follow dietary changes among the population over time, and future testing of the PHDS in different and more current datasets. Furthermore, the development of the index may be necessary based on the results of the future studies.

### Strengths and limitations

The strengths of this study include the relatively long follow-up time and harmonized and pooled data from two population-based studies: the validated FFQ and professionally measured anthropometric variables (25–27, 34). We acknowledge the limitations with the under- and overreporting and memory bias related to the FFQ method. Consequently, we conducted sensitivity analyses excluding energy under-reporters and those of reporting any possible cofounding health problems (37, 38). However, excluding under-reporters does not necessarily exclude participants that flatter their dietary intake, which may have affected their overall diet quality and attenuated associations between diet quality and anthropometric changes. We also used two different dietary indices for analyzing the association between overall diet quality and anthropometric changes. Even though the indices differ by the number of variables included (uRFDS 9, PHDS 13), the content they are assessing, and the scoring systems, the results were similar in this study population.

There are some limitations in this study. There might have been some selection bias among those who participated in the follow-up studies, which may limit generalizing our results to some extent (42). For example, those who did not participate in the follow-up were analyzed to be older, lower educated, and more physically inactive in their leisure-time but less often smokers compared to those who attended. Furthermore, those who did not participate in the follow-up tended to have higher BMI and WC at the baseline, which could have attenuated the associations between diet quality and anthropometric changes. Furthermore, related to developing the PHDS for Finnish food culture, we based the scoring system partly on the previous studies for the EAT-*Lancet* score (13) and the World Index for Sustainability and Health (WISH) (31). We are, however, familiar with the criticism for binary scoring of the EAT-*Lancet* score that could lead to good points for unhealthy diets (43). When developing our index, we tried to take this into account by simultaneously considering each component’s environmental impact. For example, as we chose the cut-off value of ≤100g/day for fish and fish products, it led to decreasing mean daily intakes according to the total PHDS. However, these choices were supported by the significant trend found between the PHDS score and index components. However, the testing of the PHDS’ plausibility against environmental aspects is beyond the scope of this study and requires further examination.

Even though the PHD has been criticized for not being affordable, for example, for people in low-income countries, and therefore not directly considering all of the Food and Agriculture Organization’s (FAO) aspects of sustainability (environmental, nutritional, economic, and sociocultural), it is the first global attempt to define clear targets to ensure the same possibilities for survival for all now and in the future (12, 44, 45). Therefore, the PHD should be regarded as a starting point for the improvement not only of the national FDGBs worldwide but also in the Nordic countries including Finland as previously environmental aspects of the diets were considered only as a distinct chapter in the FNR 2014 (11, 29, 46, 47). The FoodMin project, which examined the climate impacts of Finnish diets, found that by reducing meat consumption to one-third of the current Finnish diet (the closest scenario to the PHD), the climate impact would decrease by 19% (48). However, as the adherence to the PHD is relatively low in the Finnish adult population, even reaching the FNR 2014 would be beneficial both for human and environmental health. According to the FoodMin project (48), diets following the FNR 2014 the most (especially, the meat consumption was decreased to 500g per week as recommended in the FNR 2014) already reduced the climate impact by 13% from the current average Finnish diet (49).

## Conclusion

In this population-based longitudinal study, no associations between the overall diet quality and anthropometric changes were found, independent of energy intake. As energy intake is one of the key factors in weight management, excluding it may have attenuated our findings. Our findings could also be explained by the low adherence to dietary indices and narrow score ranges, for which detecting differences between participants was infeasible. As knowledge on the environmental aspects of nutrition has been rapidly rising in recent years, further research is needed with more recent dataset.
