# Supplementary material for: Associations of EAT-Lancet Planetary Health Diet or Finnish Nutrition Recommendations with changes in obesity measures: a follow-up study in adults
Source: Food Nutr Res. 2023 Dec 1;67:10.29219/fnr.v67.9107. doi: 10.29219/fnr.v67.9107 (PMC10710861; doi:10.29219/fnr.v67.9107)
Supplement: Supplementary file 1 [file FNR-67-9107-s001.docx]

**HEALTH 2000**

- Carried out in 2000–2001
- Population based sample in two stages
  - 1^st^: 80 out of 249 health care

districts in mainland Finland based on five university hospital regions were chosen

- - 2^nd^: a random sample was drawn from within the 80 districts
- A total of 9922 men and women, aged

≥ 18 y, were invited

- - Of these, 8028 were ≥ 30 y and

were invited to health examination

- - - **6771** (84%) in total participated in health examination

**FINRISK 2007**

- Population based sample from five geographical regions: Capital metropolitan, Southwestern Finland, North Karelia, Northern Savo, and Ostrobothnia
- A total of 9958 men and women, aged 25-74 y, were invited to participate from January to March in 2007.
  - Of which a total of **6258** participated

**DILGOM 2007**

- All participants from FINRISK 2007 were invited to participate from April to June in 2007

**° 5024** (80%) men and women participated

**HEALTH 2011**

- Carried out in 2011–2012
- All participants (alive, address available, living in Finland, and not refused to attend) from Health 2000 sample were invited
  - In total 6319, aged ≥ 41 y were invited
    - 4006 (63%) participated to health examination
      - Of which **3903** participated health examination both in Health 2000 and 2011

**DILGOM 2014**

- All participants (alive, address available, living in Finland, and not refused to attend) from baseline DILGOM study were invited to participate from April to June in 2014 (n = 4581)
  - of invited **3735** (82%) men and women participated in two groups
    - Group 1 (health examination) (n = 1312)
    - Group 2 (questionnaires and measuring tape) (n = 2423)

**CURRENT STUDY**

**(n_total_ = 4624; Health 2000/2011 = 3432 and DILGOM 2007/2014 = 1192)**

- All participants with baseline information on FFQ and baseline and follow-up information on weight, BMI and waist circumference measured by trained research nurses were included
  - pregnant participants were excluded (Health 2000 n = 28, Health 2011 n = 0, DILGOM 2007 n = 27, DILGOM 2014 n = 2)
  - participants <30 y at the baseline or ≥81 y at the follow-up were excluded (Health 2011 n = 152, DILGOM 2007 n = 85 )
  - participants with daily energy intake values < 600 and > 7000 kcal were excluded in Health 2000 (n = 18), and participants whose daily energy intake corresponded to 0.5% at either end of the energy intake distribution were excluded in DILGOM 2007 (n = 48 )

Supplemental figure 1. Flowchart of the included studies (Health 2000 and 2011, DILGOM 2007 and 2014).

Supplemental table 1. The EAT-Lancet Planetary Health Diet adapted for Finnish culture (Planetary Health Diet Score).

| **Dietary components**  **in the index** | **Original**  **EAT-*Lancet* recommendation^1^** | **Classification^2^**  Healthiness  Environmental | **Scoring** | **Reasoning for scoring** |
| --- | --- | --- | --- | --- |
| **Whole grains**  *incl. rye, oats, barley* | 232 g/d  (0-60 E%) | Protective  Low | ≥ 232 g -> 1p  < 232 g -> 0p | Rye, oats, and barley were chosen to reflect whole grains in the Finnish diet.  232 g and over was chosen to avoid same scoring for almost zero consumption and the higher consumptions. Whole grains are beneficial for health and therefore encouraged to be consumed, as well as whole grains have low impact on environment, therefore higher than 232 g consumption rates are considered less detrimental for environment. |
| **All vegetables**  *leafy vegetables, fruit vegetables, cabbages, mushrooms, legumes, and roots (excl. potato)* | 300 (200-600) g/d | Protective  Low | ≥ 300 g -> 1p  < 300 g -> 0p | 300 g and over was chosen as vegetables are beneficial for health and have in general a low impact on environment, therefore higher than 600g consumption rates are considered less detrimental for environment.  The lower limit of 300g was chosen to consider the typical over-reporting of vegetables; furthermore, FFQs tend to overestimate vegetable consumption^3^. |
| **Potatoes** | 50 (0-100) g/d | Neutral^4^  Low | ≤ 100 g -> 1p  > 100 g -> 0p | 100 g and lower were chosen according to the original upper limit recommendation as potatoes are an important component of the Finnish and the Nordic diet and with the neutral effect for the environment. |
| **Fruits and berries** | 200 (100-300) g/d | Protective  Low | ≥ 200 g -> 1p  < 200 g -> 0p | 200 g and over was chosen as fruits and berries (emphasizing Nordic fruits and berries) are beneficial for health and have in general a low impact on environment, therefore higher than 300g consumption rates are considered less detrimental for environment.  The lower limit of 200 g was chosen to consider the typical over-reporting of fruits and berries; furthermore, FFQs tend to overestimate vegetable consumption.^3^ |
| **Dairy foods**  *incl. milk, sour milk and cheese, cream, ice cream and butter as milk equivalents^5^* | 250 (0-500) g/d | Protective  Medium | ≤ 500 g -> 1p  > 500 g -> 0p | Upper limit of 500 g was chosen according to the original upper limit recommendation as dairy foods are currently important components in the Finnish food culture as they are the important source of calcium and vitamin D (because of the supplementation of milk products).  The dairy foods included all products despite the fat quality, therefore dairy fats were considered in the fat ratio component of the index. |
| **Red and processed meat**  *incl. beef, pork, lamb, sausage, meat products, offal* | 14 (0-28) g/d | Limit  High | ≤ 28 g -> 1p  > 28 g -> 0p | Upper limit of 28 g was chosen to emphasize flexibility of the planetary health diet and the advantageous nutrients for health (e.g., iron, vitamin B_12_) in red meat. |
| **Chicken, other poultry** | 29 (0-58) g/d | Neutral  Medium | ≤ 58 g -> 1p  > 58 g -> 0p | Upper limit of 58 g was chosen to emphasize flexibility of the planetary health diet according to the original upper limit recommendation. |
| **Eggs** | 13 (0-25) g/d | Neutral  Medium | ≤ 25 g -> 1p  > 25 g -> 0p | Upper limit of 25 g was chosen to emphasize flexibility of the planetary health diet according to the original upper limit recommendation. |
| **Fish**  *incl. fish, fish products, shellfish* | 28 (0-100) g/d | Protective  High | ≤ 100 g -> 1p  > 100 g -> 0p | Upper limit of 100 g was chosen to emphasize flexibility of the planetary health diet and advantageous nutrients for health in the fish. A fourfold upper limit compared to the red and processed meat emphasize the protective impact of the fish consumption for health.  Furthermore, in the Finnish food culture the natural freshwater fish species is recommended. |
| **Legumes**  *dry beans, lentils, peas, soy foods* | 75 (0-150) g/d | Protective  Low | ≥ 75g -> 1p  < 75g -> 0p | 75 g and higher was chosen to avoid same scoring for zero consumption and the higher consumptions. Legumes are beneficial for health and have a low impact on environment, therefore higher than 150 g consumption rates are not that detrimental for environment.  By setting the limit to the 75 g or higher, the transformation of the food system to more plant-based direction was emphasized. |
| **Nuts and seeds** | 25 (0-75) g/d | Protective  Medium | ≥ 25g -> 1p  < 25g -> 0p | 25g and higher were chosen to avoid same scoring for zero consumption and the higher consumptions, even though nuts have a medium impact on environment. Furthermore, in Finnish food culture the consumption rates of nuts and seeds are currently very low.  By setting the limit to the 25 g or higher, the transformation of the food system to more plant-based direction was emphasized. |
| **Ratio of unsaturated and saturated fat intake**  *MUFA + PUFA /*  *SFA + trans-FA* | Unsaturated oils  40 (20-80) g/d  Palm oil  6.8 (0-6.8) g/d  Dairy fats  (incl. in milk)  0 g/d  Lard or tallow  5 (0-5) g/d | Unsaturated fats:  Protective  Low  Saturated fats:  Limit  High | ≥ 2.0 -> 1p  < 2.0 -> 0p | The ratio of unsaturated and saturated fats was chosen to measure the quality of the fat in the diet instead of using only unsaturated fat intake.  The ratio of 2,0 were chosen according to the Nordic nutrition recommendation; 2/3 of total fat from unsaturated fats and 1/3 of total fat from saturated fats resulting ratio of 2,0. |
| **Sucrose**  *total sucrose intake* | All sweeteners  31 (0–31) g/d | Limit  Low | ≤ 31 g -> 1p  > 31 g -> 0p | Total sucrose intake was used as a surrogate for added sugar intake.  31 g or lower consumption of all sweeteners were chosen according to the original recommendations. |

g/d, grams/day; incl. = included; MUFA, monounsaturated fatty acids; PUFA, polyunsaturated fatty acids; SFA, saturated fatty acids; *trans*-FA, *trans* fatty acids

^1^ Based on Willet et al. Food in the Anthropocene: the EAT-Lancet Commission on healthy diets from sustainable food systems. Lancet 2019;393:447-92

^2^ Based on Trijsburg L, et al. Method for the Development of WISH, a Globally Applicable Index for Healthy Diets from Sustainable Food Systems classification (Table 1). Nutrients 2020;30;13(1):93.

Healthiness of the included food groups (excl. potatoes) were classified as protective, neutral, or limit (originally based on the supplementary material of the EAT-*Lancet* recommendations), and the impact on environment as low, medium, or high (originally based on the assessment of Clark, et al. Proc Natl Acad Sci USA 2019;12:(46):23357-62).

^3^ Based on FFQ validation study by Männistö S, et al. Reproducibility and Validity of a Food Frequency Questionnaire in a Case-Control Study on Breast Cancer. J Clin Epidemiol 1996; 49: 401–9.

^4^ Classified based on Clark et al. Multiple health and environmental impacts of foods. Proc Natl Acad Sci USA 2019;12:(46):23357-62. However, the healthiness of plain potatoes is not clear, as potatoes are analyzed usually with potato products, e.g., fries and chips, therefore ‘neutral’ impact on health was used here (Robertson TM, et al. Starchy Carbohydrates in a Healthy Diet: The Role of the Humble Potato. Nutrients 2018;14;10(11):1764).

^5^ Wood A, et al. Erratum: Nordic food systems for improved health and sustainability – baseline assessment to inform transformation. Stockholm Resilience Centre, Stockholm University. 2019. Available from: https://www.stockholmresilience.org/download/18.8620dc61698d96b1901719c/1561013818461/Erratum_Nordic%20report_14-6-19.pdf.

Supplemental table 2. Energy-standardized (grams per megajoules) cut-off values for scoring of each component of updated Recommended Finnish Diet Score by baseline studies (Health 2000 and DILGOM 2007).

|  | Health 2000 | | |  | DILGOM 2007 | | |
| --- | --- | --- | --- | --- | --- | --- | --- |
|  | 1^*^ | 2^^^ | 3^¤^ |  | 1^*^ | 2^^^ | 3^¤^ |
| Whole grains | 3.4 | 6.3 | 9.2 |  | 4.0 | 6.9 | 9.7 |
| Vegetables | 17.9 | 26.2 | 37.7 |  | 21.8 | 30.1 | 43.6 |
| Fruits and berries | 9.1 | 18.0 | 31.4 |  | 13.2 | 24.3 | 40.9 |
| Red and processed meat | 18.3 | 14.2 | 10.8 |  | 17.11 | 12.62 | 9.0 |
| Fish and fish products | 2.8 | 4.2 | 6.0 |  | 3.0 | 4.6 | 6.5 |
| Ratio of PUFA to SFA+trans-fatty acids | 1.1 | 1.2 | 1.3 |  | 1.2 | 1.4 | 1.6 |
| Sucrose | 6.3 | 5.0 | 3.9 |  | 6.8 | 5.5 | 4.2 |
| Salt | 1.3 | 1.2 | 1.1 |  | 1.0 | 0.9 | 0.8 |
| Alcohol | 0.8 | 0.3 | 0.1 |  | 1.3 | 0.6 | 0.2 |

Values presented as grams per megajoules.

^*^ cut-off value between 0 and 1 point

^^^ cut-off value between 1 and 2 points

^¤^ cut-off value between 2 and 3 points

|  | Weight | | | |  | BMI | | | |  | Waist circumference | | | |
| --- | --- | --- | --- | --- | --- | --- | --- | --- | --- | --- | --- | --- | --- | --- |
|  | ≤ -5.0 % | -4.9–4.9 % | 5.0–9.9 % | ≥ 10.0 % |  | ≤ -5.0 % | -4.9–4.9 % | 5.0–9.9 % | ≥ 10.0 % |  | ≤ -5.0 % | -4.9–4.9 % | 5.0–9.9 % | ≥ 10.0 % |
| n | 488 | 2957 | 795 | 384 |  | 435 | 2972 | 808 | 409 |  | 388 | 3033 | 846 | 357 |
| Women, % | 56 | 51 | 63 | 72 |  | 56 | 50 | 64 | 74 |  | 67 | 51 | 61 | 69 |
| Age, y | 55 (11) | 50 (11) | 46 (11) | 44 (10) |  | 55 (11) | 50 (11) | 46 (11) | 44 (10) |  | 54 (11) | 50 (11) | 48 (11) | 45 (11) |
| High education level^1^, % | 33 | 39 | 41 | 40 |  | 36 | 39 | 39 | 40 |  | 36 | 38 | 42 | 42 |
| Current smokers, % | 20 | 21 | 26 | 38 |  | 21 | 21 | 25 | 38 |  | 15 | 21 | 26 | 37 |
| Leisure-time physical inactivity^2^, % | 24 | 19 | 24 | 28 |  | 24 | 19 | 24 | 28 |  | 22 | 20 | 23 | 26 |
|  |  |  |  |  |  |  |  |  |  |  |  |  |  |  |
| Energy, kcal | 2277 (732) | 2307 (779) | 2339 (803) | 2269 (812) |  | 2292 (772) | 2312 (772) | 2324 (805) | 2246 (810) |  | 2245 (771) | 2330 (782) | 2285 (796) | 2226 (747) |
| Carbohydrate, E% | 45 (6.3) | 44 (5.7) | 44 (5.6) | 44 (6.3) |  | 45 (6.4) | 44 (5.7) | 44 (5.5) | 44 (6.1) |  | 45 (6.1) | 44 (5.7) | 44 (5.9) | 44 (6.0) |
| Fiber, g | 28 (12) | 26 (11) | 26 (11) | 25 (12) |  | 28 (12) | 26 (11) | 26 (12) | 25 (12) |  | 27 (12) | 26 (11) | 26 (12) | 25 (12) |
| Protein, E% | 17 (2.4) | 17 (2.3) | 17 (2.4) | 17 (2.4) |  | 17 (2.4) | 17 (2.3) | 17 (2.3) | 17 (2.4) |  | 17 (2.3) | 17 (2.3) | 17 (2.3) | 17 (2.6) |
| Fat, E% | 35 (5.7) | 36 (5.3) | 36 (5.4) | 36 (5.8) |  | 35 (5.8) | 36 (5.3) | 36 (5.3) | 36 (5.8) |  | 35 (5.4) | 36 (5.3) | 36 (5.6) | 36 (5.8) |
|  |  |  |  |  |  |  |  |  |  |  |  |  |  |  |
| Index scores^3^ |  |  |  |  |  |  |  |  |  |  |  |  |  |  |
| PHDS | 3.8 (1.3) | 3.6 (1.2) | 3.7 (1.3) | 3.6 (1.2) |  | 3.8 (1.2) | 3.6 (1.2) | 3.7 (1.2) | 3.6 (1.3) |  | 3.7 (1.2) | 3.6 (1.2) | 3.6 (1.2) | 3.6 (1.3) |
| uRFDS | 13.9 (3.8) | 13.5 (3.8) | 13.5 (3.8) | 13.0 (3.7) |  | 13.8 (3.8) | 13.5 (3.7) | 13.5 (3.8) | 13.3 (3.7) |  | 14.1 (3.9) | 13.5 (3.7) | 13.3 (3.8) | 13.3 (3.9) |
|  |  |  |  |  |  |  |  |  |  |  |  |  |  |  |
| Weight, kg | 80.3 (16.4) | 76.8 (14.5) | 75.0 (14.9) | 72.7 (15.0) |  | 80.9 (16.5) | 76.8 (14.4) | 74.8 (15.5) | 72.9 (14.5) |  | 78.0 (15.7) | 77.0 (14.9) | 75.3 (14.6) | 72.9 (14.4) |
| BMI, kg/m^2^ | 28.3 (4.9) | 26.5 (4.3) | 26.2 (4.5) | 25.7 (4.5) |  | 28.5 (5.0) | 26.4 (4.2) | 26.3 (4.6) | 25.8 (4.5) |  | 27.8 (4.9) | 26.6 (4.5) | 26.3 (4.2) | 25.8 (4.4) |
| Waist circumference, cm | 96.1 (13.6) | 91.4 (12.6) | 89.2 (13.0) | 87.2 (12.9) |  | 96.4 (13.6) | 91.3 (12.5) | 89.7 (13.5) | 87.3 (12.5) |  | 95.5 (13.0) | 91.7 (12.9) | 89.3 (12.5) | 85.9 (12.0) |

Supplemental table 3. Background and dietary characteristics of the participants of pooled surveys (Health 2000 and DILGOM 2007) at baseline by categorised percentual changes of weight, body mass index and waist circumference presented as means with standard deviations or percentages.

E%, percentage of total energy intake; BMI, body mass index; PHDS, Planetary Health Diet Score; uRFDS, updated Recommended Finnish Diet Score

Anthropometric changes were harmonized between the studies by dividing the change by the follow-up years (7 or 11) and then multiplied by 7 to standardize changes.

Weight, BMI, or waist circumference changes were categorized to four groups: weight loss if percentual change was -5.0 % or less, weight stable if percentual weight change was between -4.9 % and +4.9%, and weight gain divided to two groups (if percentual change was 5.0% or more but less than 10.0%, and weight gain if weight change was 10% or more).

^1^ Health 2000: graduated from university or university of applied sciences; DILGOM: highest tertile of self-reported total school years according to birth cohort to adjust for the extension of the basic education system and the increase of average school years over time

^2^ Leisure-time physical inactivity; light activities, like reading and watching television

^3^ PHDS ranged from 0 to 13 points and uRFDS ranged from 0 to 27 points.

Supplemental table 4. Pooled energy-standardized (2500 kcal/d) daily intakes of nutrients and Planetary Health Diet Score components of the participants of pooled studies (Health 2000 and DILGOM 2007) by the Planetary Health Diet Score presented as means with standard deviations.

|  | Planetary Health Diet Score | | | | | | | | | |  |  |
| --- | --- | --- | --- | --- | --- | --- | --- | --- | --- | --- | --- | --- |
|  | 0 | 1 | 2 | 3 | 4 | 5 | 6 | 7 | 8 | 9 | β (95 % CI) | p* |
| n | 4 | 71 | 731 | 1392 | 1421 | 738 | 202 | 46 | 13 | 4 |  |  |
| Carbohydrate, E% | 38 (4.2) | 40 (5.1) | 43 (4.7) | 43 (5.3) | 45 (5.8) | 46 (6.1) | 47 (6.9) | 47 (7.1) | 47 (8.6) | 48 (7.5) | 0.86  (0.73, 1.00) | < 0.001 |
| Fiber, g | 24 (5.6) | 22 (5.1) | 24 (6.3) | 26 (7.4) | 30 (8.2) | 34 (8.8) | 36 (11) | 41 (10) | 47 (12) | 57 (6.8) | 2.83  (2.65, 3.01) | < 0.001 |
| Protein, E% | 20 (2.0) | 19 (1.9) | 17 (2.0) | 17 (2.3) | 17 (2.4) | 17 (2.4) | 17 (2.6) | 16 (3.1) | 16 (2.2) | 15 (1.2) | -0.29  (-0.66, 0.08) | 0.12 |
| Fat, E% | 40 (4.5) | 39 (5.1) | 38 (4.4) | 37 (5.0) | 35 (5.4) | 34 (5.6) | 33 (5.8) | 33 (6.3) | 32 (9.2) | 34 (8.0) | -0.85  (-1.06, -0.63) | < 0.001 |
|  |  |  |  |  |  |  |  |  |  |  |  |  |
| Whole grains, g | 72 (51) | 52 (32) | 62 (38) | 68 (40) | 73 (42) | 81 (45) | 88 (59) | 91 (64) | 78 (41) | 65 (42) | 0.06  (0.05, 0.08) | < 0.001 |
| Vegetables, g | 251 (17) | 206 (90) | 211 (97) | 258 (159) | 330 (180) | 416 (202) | 450 (229) | 508 (270) | 581 (269) | 777 (563) | 0.15  (0.08, 0.21) | < 0.001 |
| Potatoes, g | 164 (54) | 209 (114) | 192 (86) | 190 (103) | 177 (108) | 168 (116) | 129 (109) | 121 (104) | 85 (76) | 51 (18) | -0.11  (-0.16, -0.06) | < 0.001 |
| Fruits and berries, g | 112 (37) | 127 (70) | 132 (97) | 201 (185) | 304 (228) | 402 (260) | 383 (244) | 461 (251) | 518 (377) | 1111 (292) | 0.23  (0.17, 0.30) | < 0.001 |
| Dairy foods, g | 881 (289) | 872 (323) | 920 (318) | 935 (341) | 936 (378) | 925 (380) | 823 (460) | 733 (360) | 642 (478) | 276 (134) | -0.05  (-0.10, -0.004) | 0.03 |
| Red and processed meat, g | 175 (53) | 159 (52) | 162 (55) | 156 (64) | 144 (66) | 134 (66) | 115 (76) | 90 (79) | 77 (106) | 98 (74) | -0.11  (-0.17, -0.06) | < 0.001 |
| Poultry, g | 91 (10) | 71 (34) | 37 (34) | 37 (37) | 37 (38) | 34 (31) | 34 (43) | 43 (83) | 20 (15) | 11 (16) | -0.12  (-0.23, -0.01) | 0.048 |
| Eggs, g | 38 (8) | 43 (20) | 37 (18) | 31 (22) | 26 (16) | 21 (11) | 21 (19) | 20 (13) | 16 (15) | 18 (4) | -0.18  (-0.19, -0.16) | < 0.001 |
| Fish, g | 128 (30) | 80 (55) | 53 (37) | 50 (33) | 50 (33) | 52 (29) | 52 (32) | 43 (26) | 37 (26) | 43 (28) | -0.05  (-0.07, -0.03) | < 0.001 |
| Legumes, g | 19 (17) | 14 (10) | 12 (9) | 13 (11) | 15 (20) | 17 (19) | 28 (55) | 66 (131) | 133 (272) | 105 (53) | 0.08  (0.06, 0.11) | < 0.001 |
| Nuts and seeds, g | 0.7 (1.3) | 2.3 (4.0) | 1.5 (2.8) | 1.6 (3.8) | 2.4 (5.6) | 3.9 (9.4) | 7.9 (18) | 18 (22) | 30 (47) | 49 (53) | 0.12  (0.05, 0.18) | < 0.001 |
| Fat ratio | 1.45 (0.29) | 1.34 (0.20) | 1.21 (0.21) | 1.22 (0.24) | 1.27 (0.26) | 1.33 (0.28) | 1.44 (0.40) | 1.74 (0.57) | 2.04 (0.73) | 2.33 (0.70) | 0.05  (0.02, 0.08) | 0.002 |
| Sucrose, g | 41 (13) | 50 (13) | 55 (16) | 55 (19) | 56 (20) | 56 (22) | 56 (26) | 58 (27) | 63 (30) | 59 (26) | -0.003  (-0.04, 0.04) | 0.867 |

SAFA, saturated fatty-acids

In the pooled data Planetary Health Diet Score scores ranged from 0 to 11 points (total score could range from 0 to 13 points), however, 10 and 11 points were received by only one person each and therefore data is not shown.

* Pooled p-values for the association between individual log-transformed (except Fat ratio) dietary score components with PHD score were determined with a two-staged random effects linear regression model adjusted for age, sex, education, leisure-time physical activity and smoking status. Significance level was at p < 0.05.

Supplemental table 5. Mean index scores with standard deviations according to sex, education level, and smoking habits.

|  | Sex | | Education^*^ | | Smoking habits | |
| --- | --- | --- | --- | --- | --- | --- |
|  | Men | Women | High education | Low education | Current smokers | Non-smokers |
| n | 2067 | 2557 | 1792 | 1201 | 1055 | 2491 |
| PHDS | 3.4 (1.1) | 3.8 (1.2) | 3.8 (1.2) | 3.5 (1.1) | 3.4 (1.1) | 3.7 (1.2) |
| uRFDS | 12.1 (3.5) | 14.6 (3.6) | 13.9 (3.7) | 13.4 (3.8) | 12.4 (3.5) | 14.0 (3.8) |

PHDS, Planetary Health Diet Score; uRFDS, updated Recommended Finnish Diet Score

^*^Education level: Health 2000: low (did not graduate from upper secondary school or vocational school) and high (graduated from university or university of applied sciences); DILGOM: the lowest or highest tertile of the total number of school years according to sex and birth cohort to adjust for the extension of the basic education system and increase in average school years over the last decades
